# 2430: Leading diverse and emerging scientists to success (LEADS)

**DOI:** 10.1017/cts.2017.180

**Published:** 2018-05-10

**Authors:** Doris Rubio, Marie Norman, Todd Seto, Alexander Quarshie, Magda Shaheen, Stephanie Bailey, George Perry, Lourdes Soto

**Affiliations:** University of Pittsburgh, Pittsburgh, PA, USA

## Abstract

OBJECTIVES/SPECIFIC AIMS: To diversify the biomedical research workforce by training postdoctoral scholars and junior faculty from 6 Minority Serving Institutions (MSIs) on practical research skills such as Critical and Creative Thinking, Formulating the Problem, Asking the Right Question, Grant Writing, and Team Science METHODS/STUDY POPULATION: In collaboration with our partners, we identified 11 topics where trainees lack research funding. Next, we identified instructors for these topics. We converted the topics to online module with modules ranging from 2 to 8 weeks. In working with an online education expert, we developed innovative online training using Moodle as the content management system. Scholars complete readings, videos, self-assessments and participate in discussion board each week. In addition, we have weekly synchronous sessions for each module. All scholars are required to take the grant writing module and 8 other modules. After each module, trainees complete a brief survey to evaluate the module. The leaders at the MSI participated in an intensive face-to-face training session on how to be a career coach so that they could be career coaches for the LEADS Scholars at their home institutions. RESULTS/ANTICIPATED RESULTS: In the first year, we selected 13 LEADS Scholars. All but 3 scholars elected to take every module. The 3 scholars did not enroll in the Peer Reviewing module. Results of the brief survey at the end of each module indicate that the scholars value each of the modules and rate them very highly. When 1 scholar wanted to leave the program, we decided to have a conference call with all of the LEADS Scholars to determine what was working and what was not working with the program. All scholars recognized the value of LEADS. Some scholars felt that the weekly synchronous session was too demanding as they have competing demands on their time. We consulted with the leadership at the MSI and decided to modify the requirements of the program such that every synchronous call was not required for successful completion of the module and to earn a badge. Scholars need to have at least 9 badges to earn a certificate. In addition to the training, we decided that scholars would also benefit from mock reviews of their grants. This will help them submit successful grants. We learned that the best way to serve the needs of the scholars is to work iteratively with the scholars and leadership to develop a successful program that most effectively meets their needs of the scholars and helps them launch a successful career. DISCUSSION/SIGNIFICANCE OF IMPACT: Postdoctoral scholars and junior faculty from MSI need practical research training to help launch their research career. We suspect that this is true of many institutions and plan to develop these modules so that they can be widely disseminated to other institutions.

